# The influence of education on performance of adults on the Clock Drawing Test

**DOI:** 10.1590/1980-57642018dn12-010009

**Published:** 2018

**Authors:** Ísis Franci Cavalcanti de Noronha, Simone dos Santos Barreto, Karin Zazo Ortiz

**Affiliations:** 1 Speech and Language Therapist. Universidade Federal de São Paulo; 2 Adjoined Professor. Department of Specific Training in Speech, Language and Hearing Sciences - Universidade Federal Fluminense, RJ, Brazil; 3 Associate Professor. Department of Speech, Language and Hearing Sciences - Universidade Federal de São Paulo

**Keywords:** ideomotor praxis, educational level, cognition, clock drawing, apraxia ideomotora, escolaridade, cognição, desenho do relógio

## Abstract

The Clock Drawing Test (CDT) is an important instrument for screening individuals suspected of having cognitive impairment.

**Objective:**

To determine the influence of education on the performance of healthy adults on the CDT.

**Methods:**

A total of 121 drawings by healthy adults without neurological complaints or impairments were analysed. Participants were stratified by educational level into 4 subgroups: 27 illiterate adults, 34 individuals with 1-4 years of formal education, 30 with 5-11 years, and 30 adults with >11 years' formal education. Scores on the CDT were analyzed based on a scale of 1-10 points according to the criteria of Sunderland et al. (1989).¹ The Kruskal-Wallis test was applied to compare the different education groups. Tukey's multiple comparisons test was used when a significant factor was found.

**Results:**

Although scores were higher with greater education, statistically significant differences on the CDT were found only between the illiterate and other educated groups.

**Conclusion:**

The CDT proved especially difficult for illiterate individuals, who had lower scores. These results suggest that this screening test is suitable for assessing mainly visuoconstructional praxis and providing an overall impression of cognitive function among individuals, independently of years of education.

## RESUMO

O Teste do Desenho do Relógio (TDR) é um instrumento importante para rastrear indivíduos com suspeita de alterações cognitivas.

**Objetivo:**

Verificar a influência da variável sociodemográfica escolaridade no desempenho de adultos saudáveis no TDR.

**Métodos:**

Foram analisados os desenhos de 121 adultos saudáveis sem queixas e/ou alterações neurológicas, distribuídos em 4 subgrupos, a partir de quatro níveis de escolaridade: 27 adultos analfabetos, 34 adultos com 1-4 anos de estudo formal, 30 adultos com 5-11 anos de estudo formal, 30 adultos com >11 anos de estudo formal. Os desenhos obtidos foram analisados segundo os critérios propostos por Sunderland et all (1989),¹ com uma escala que variou de 1 a 10 pontos. A comparação dos grupos segundo a escolaridade foi feita utilizando-se o teste de Kruskal-Wallis seguido do teste de comparações múltiplas de Tukey para verificar onde as diferenças se deram.

**Resultados:**

Apesar dos escores terem aumentado conforme a escolaridade, foram observadas diferenças estatisticamente significantes apenas entre analfabetos e os demais grupos no TDR.

**Conclusão:**

O desempenho dos indivíduos melhorou de acordo com a escolaridade, porém o TDR não sofreu forte influência da escolaridade em sua aplicação, exceto para indivíduos analfabetos, que apresentaram escores diminuídos. Deste modo, acredita-se que o teste pode ser utilizado na avaliação da praxia visuo-construtiva em indivíduos independentemente dos anos de escolarização.

The Clock Drawing Test (CDT), Mini-Mental State Exam (MMSE) and functional scales have been widely used in research protocols and clinical settings for screening individuals suspected of having cognitive impairment^2^. The CDT consists of a clock drawing task, including the dial, numbers and hands which have to be drawn and set to the time requested by the examiner. The test is designed to assess executive functions (planning, logical sequence, abstraction ability and execution monitoring), visuospatial organization, visuoconstructional praxis, psychomotor coordination and working memory.^1^ As a test involving several cognitive functions, it is indicated for providing an overall impression of cognitive function of an individual. The CDT is not a diagnostic test but has been used alone or in association with other tests^3^^,^ 4 to screen for cognitive deficits,^5^ particularly in elderly people.^6^

Demands of the CDT of the subject being assessed include auditory comprehension of the command given,^4^^,^ 5 concentration and attention in performing the drawing,^2^^,^ 4 ^,^ 6 the use of numeric memory,^7^ semantic memory (to remember what a clock is and what it looks like)^8^ and episodic memory (to recall all elements of the command given).^8^^,^ 9

Visuospatial praxis is used to identify and understand the meaning of images in the visual field (understanding the meaning of elements being drawn) and for handling and reproducing spatial information (reproducing the image of a clock with its numbers and hands placed correctly within the space).^8^^-^ 10 Executive functions are also required and fundamental, given that they aid the executive of complex tasks or behaviors, even in the presence of irrelevant, new or ambiguous stimuli. They are involved in motivation for executing tasks, in their planning (e.g. the order in which the clock elements are drawn), in monitoring response (ability to identify errors, carry out corrections or deploy new strategies in the event that problems arise during execution).^2^^,^ 11

Education has been identified as a sociodemographic variable which predicts the neuropsychological performance of healthy individuals on tasks assessing cognitive functions.^12^ Therefore, it is essential to adjust test scores according to individuals' educational level in order to prevent unfamiliarity or cultural limitations from being interpreted as deficits.^13^

Studies conducted in recent years have shown a strong effect of education on language and cognitive abilities of healthy individuals.^14^^,^ 15 Given that the CDT assesses cognitive functions such as visuoconstructional praxis, working memory and executive/planning functions, and that these are influenced by education, it is hypothesized that several of these abilities are improved with formal learning.

For many assessment instruments used in Brazil, normative data are drawn from studies involving populations of developed countries, not accommodating the variable educational levels found in the Brazilian population.^16^ The CDT has been widely used and extensively studied in Brazil,^17^^,^ 18 but most studies have investigated the test and its scoring system.^19^^,^ 20 Studies reporting the effects of education on performance on this widely used instrument were also found, predominantly involving elderly subjects.^21^^,^ 22 Considering that the CDT can be used in different populations, the objective of the present study was to determine the influence of education on the performance of healthy adults on the CDT.

## METHODS

The study was approved by the local Research Ethics Committee of the Federal University of São Paulo (protocol number 0113/2016) . After receiving full information about the study, written informed consent was obtained from all enrolled subjects.

### Participants

A retrospective study analyzing the data of 121 healthy adults aged 19-59 years was carried out. The volunteers were selected from individuals accompanying patients of the Acquired Speech and Language Neurological Disturbances outpatient unit and other clinics within the Department of Speech, Language and Hearing Sciences, Unifesp. Inclusion Criteria: attainment of normal scores on the MMSE cognitive screening tests^23^ and Phonemic Verbal Fluency (PVF) test.^24^ Exclusion criteria: individuals with a history of current or previous neurologic or psychiatric diseases, uncontrolled systemic diseases, self-reported communication disturbances, complaints of cognitive difficulties, use of psychotrophic medication, history of alcohol abuse or use of illegal drugs, uncorrected visual or auditory deficits that may affect test performance, more than one repeat on school history or lower-than-expected performance (cut-offs for normality) on cognitive screening tests.

Participants were divided into 4 subgroups, pooled and then analyzed according to four educational levels (as defined for the Brazilian education system): [1] 27 illiterate adults with 0 years of formal education; [2] 34 adults with 1-4 years; [3] 30 adults with 5-11 years; and [4] 30 adults with >11 years of formal education. All patients were assessed by two researchers with expertise in neuropsychological tests.

### Procedures

The results on the MMSE^25^ were analyzed based on norms for the Brazilian population obtained in the study by Brucki et al. (2003).^23^ The minimum values determined for the population assessed by the cited authors were employed. For illiterate subjects: score of 11; individuals with 1-4 years' education: score of 16; 5-8 years' education: score of 19; 9-11 years' education: score of 22; and >11 years' education: score of 22.

For the PVF assessment, the FAS was previously applied, whose letters are amongst the most commonly occurring in Brazilian Portuguese.^24^ Speakers were asked to produce as many words as possible in sixty seconds, beginning with each of the three letters uttered by the examiner. The letters were given in the following order: F, A and S. Participants were told which words would not be scored: repeated words, proper nouns, derived words differing only by number, gender, degree or conjugation. The test was applied and scored according to the criteria established by Senhorini et al. (2006).^24^ Utterances of each volunteer were sound recorded and orthographically transcribed.

The number of valid words produced were tallied for each task and the total words produced on the three tasks was calculated, giving four scores per volunteer (number of words beginning with F, number of words beginning with A, number of words beginning with S and total number of words beginning with F, A and S). Given the high reliability of scores derived from verbal fluency tasks reported in the Brazilian literature, no procedures analyzing inter-examiner or test-retest concordance were applied.^26^

The data from participants considered eligible for the study were subjected to analysis and scoring on the CDT. For the CDT, participants were given a blank sheet of paper with a pre-drawn circle placed center page and asked to draw the face of an analogic clock, with placement of all numbers and hands set to the time of ten minutes past eleven. Scores were analyzed based on a scale of 1-10 points according to the criteria of Sunderland et al. (1989).^1^ The maximum score of ten points was given to drawings representing the hands in the correct position; nine points for slight errors in the placement of hands; eight points for more noticeable errors in hands; seven points for hands significantly off course; six points for inappropriate or alternative forms of showing the time requested; five points for crowding of numbers in the first or last quadrant, reverse order in placement of the numbers on the clock face, but with hands present in some fashion; four points in the case of distortion of number sequence of the clock, with lost integrity of the clock face (numbers outside the clock face, numbers missing); three points for hands not present; two points for a vague representation of a clock; and one point for absence of the drawing or sketch of a graphical image.

### Statistical analysis

The Kolmogorov-Smirnov normality test was applied to determine whether the data obtained fitted the curve for normality (Gauss curve). The data did not display a pattern of normality and therefore the Kruskal-Wallis test was applied to compare the different education groups. Tukey's multiple comparisons test was used when a significant factor was found.

A probability (p) of less than 0.05 was considered statistically significant.

All of the calculations were performed using the statistical software SPSS (Statistical Package for the Social Sciences) 13.5.1 for Windows.

## RESULTS

Of the 121 participants, 63 were women (52.1%) and 58 were men (47.9%). Mean age of the population analyzed was 39.6 years (SD = 11.9) and mean performance on the MMSE was 27.2 (SD 2.9) points, within the limits of normality expected for educational level.

The results obtained by the population analyzed in this study on the CDT are given below.

Table 1 depicts the means and standard deviations on the CDT according to educational level together with the results on the Kruskall-Wallis test for group comparison.

**Table 1:** Means and standard deviations on CDT according to educational level.

	Educational Level				Kruskall-Wallis
	Illiterate	1-4 years	5-11 years	>11 years	Test (p)
Minimum	0	4	2	8	
Maximum	10	10	10	10	
Mean	5.74	8.32*	8.13*	9.23*	
Median	5.00	10.00	8.50	9.50	<0.001*
Standard deviation	3.526	2.383	1.776	1.104	

* Significant difference on comparison of means by Kruskall-Wallis test at probability level of 0.1% (p<0.001).

**Figure 1:**
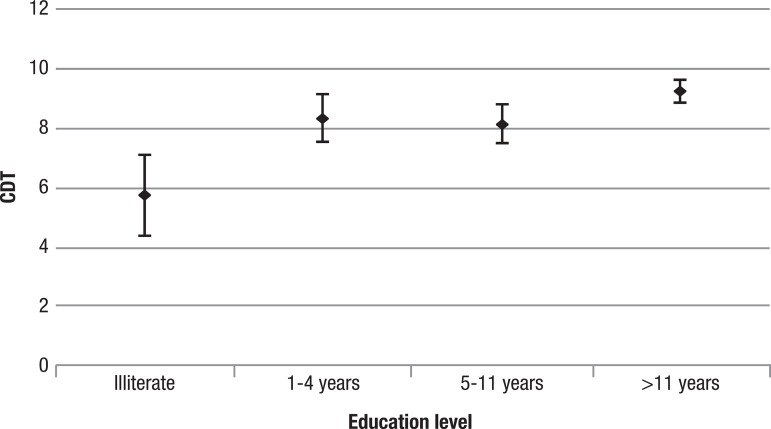
CDT scores for the groups investigated. Confidence interval for mean: mean ± 1.96. *standard-deviations / √ (n-1).

For improved visualization of the results, the scores obtained by the groups on the CDT are depicted in Figure 1.

The greatest difference observed is between the illiterate group and the other educated groups.

Tukey's multiple comparisons test was performed to determine where the differences occurred.

Results of the performance comparison among groups are given in Table 2.

**Table 2:** Results on Tukey's multiple comparisons test for groups with different educational levels.

	Illiterate (p)	1-4 years (p)	5-11 years (p)
1-4 years	<0.001*		
5-11 years	<0.001*	0.988	
>11 years	<0.001*	0.408	0.267

* Significant difference on comparison of means by Tukey's test at probability level of 0.1% (p<0.001).

As shown in Table 2, there was no significant difference among the groups exposed to formal education (1-4 year X 5-11 years X >11 years of formal education), although a significant difference was found between all the educated groups and the illiterate group.

## DISCUSSION

The most prominent finding of this study was the absence of difference among the groups exposed to formal education on comparison of performance according to different educational levels. However, a difference was found between educated individuals and illiterate subjects.

In countries that are more socioeconomically developed than Brazil, the minimum level of education is five years.^12^ Nevertheless, given the educational profile of the Brazilian population, the present study sample comprised individuals with heterogeneous levels of education, including illiterate subjects.

Although mean scores were progressively higher among groups exposed to greater formal education, 1-4 years (mean 5.74 and SD 3.53); 5-11 (mean 8.32 and SD 2.38) and >11 years (mean 8.13 SD 1.78), these differences were not statistically significant. In a previous study assessing several cognitive domains of individuals with 0-22 years of formal education, in order to obtain a very reliable brief neuropsychological assessment battery, the authors noted that the education effect was non-linear: significant differences were evident between groups with 0-3 versus 10-22 years of formal education, whereas the same was not observed between groups with 4-9 and 10-22 years of formal education. In addition, no difference in attention and orientation domains were found between individuals with ≥4 years of education and those with ≤9 years of education, who had similar performance for memory.^27^ This data is very important since it suggests that formal education does not impact all the cognitive domains in the same way. Although several studies have previously demonstrated the influence of education on CDT results,^22^ most of these analyzed subjects with at least four years of formal education or elderly subjects.^17^^,^ 21 ^,^ 22 In the present study, although differences were found among groups, with better scores for higher education, no statistical differences were found. It is noteworthy that only adults were evaluated in the present study. This might explain the difference found between the present study results and those of previous investigations, since older participants can have worse performance on cognitive tasks.^28^^,^ 29 No previous studies investigating the performance of illiterate normal subjects on the CDT were found.

Another study^30^ assessed the cognitive performance of adults and active elderly for different levels of education. The CDT was used to assess executive functions and revealed no differences between groups with different educational level for the domains of planning and sequencing. Likewise, the results of the present study showed similar performance for different levels of education.

Individuals with 1-4 years of formal education had similar performance to the other groups, although studies performed on other cognitive functions showed performance differences between low educated individuals (1-4 years) and those with 8-11 years and >11 years of formal education.^15^^,^ 24 ^,^ 31 A possible hypothesis is that elementary school years may have promoted the development of the abilities required to process the information needed to produce the drawing, evident at 1-4 years' education. This may be the case because, during the pre-literacy period, many abilities involved in the clock drawing such as visuoconstructional praxis, visuospatial organization and psychomotor coordination, are stimulated. Acquisition and refinement of motor skills and the first movement combinations, which allow the development of global and fine motor skills, whereby children considerably increase their motor repertoire and acquire the models of movement coordination essential for subsequent skilled performances.^32^ In a previous study,^22^ a qualitative analysis of the CDT by educational level was performed, revealing significant differences in quantitative and qualitative CDT scores between educational levels. The qualitative analysis added information about the pattern of errors committed. Errors in numbers display, numbers in the counterclockwise direction, incorrect representation of the clock, and mild graphic difficulties were prominent in the lower education group. These errors indicated spatial, planning and conceptual deficits and the participants with lower education seemed to have more difficulties with respect to the meaning, interpretation and knowledge of the clock.^22^

Education influences cognitive screening tests^30^ and individuals with higher levels of education perform better on tests assessing processing speed, attention, executive functions, memory and intelligence.^33^ However, everyday use of the clock may have helped in the production of the drawing, explaining the absence of difference among the groups exposed to formal education.

The difference among educated and illiterate individuals corroborates studies comparing performance on neuropsychological tasks. The findings of these studies show better performance by educated than uneducated subjects.^25^

A number of studies investigating different cognitive functions reveal statistically significant disparities between groups with different levels of education on linguistic tasks such as oral comprehension, reading, graphical comprehension, naming, lexical availability, dictation and written naming of actions^15^ as well as on language tasks involving semantic knowledge, judgement of traits and naming, besides tasks of calculus,^31^ memory^17^ and orofacial praxis.^14^ In the present study, no difference among educated groups was evident, but it is noteworthy that unlike this investigation, previous studies analyzed abilities more dependent on formal learning.

Comparing this study with previous investigations shows that the performance of the group of illiterate individuals was similar to the performance of elderly with Alzheimer due to dementia. The effect of absence of education may simulate or exceed the effect of the neurological condition.^34^ Epidemiological studies in different cultures report that persons with low educational level tend to more readily classified as having dementia.^35^ Indeed, education can represent a confounding factor in the diagnosis of dementia conditions, if isolated from other factors. The researchers used the MMSE and other gold standard measures for diagnosing dementias to compare two groups, one with formal education and one without. The group diagnosed with dementia had more zero scores on the test, followed by the group without dementia and no formal education. Among non-demented participants, the group with formal education performed better than the group without formal education throughout test. In general, participants answered the MMSE subtests correctly and the tests with most errors were registration, object naming and three commands, irrespective of dementia diagnosis or educational level. On the subtests attention, calculus, figure copying and those requiring reading abilities, participants with dementia and those without dementia and no formal education had an error rate of 90% on these items. These results showed that, despite classification for education, the MMSE had more false positive effects in individuals without formal education than the group with formal education.^36^

The CDT was not strongly influenced by education in its application, except for illiterate individuals, who attained statistically significant lower scores. Thus, the CDT can be used clinically for screening cognitive deficits irrespective of education.

The comparison of performance on the CDT among the educated groups revealed that individuals with 1-4 years and 5-11 of formal education had an average score of 8 points, warranting clinical attention given than a score of between 6 and 8 suggests suspected cognitive impairment.

In the diagnostic process of dissociations between deficits and preserved cognitive abilities, the effect of individual factors can lead to false positive results, such as when a low educational level can be associated with a lower-than-expected performance, resembling the performance secondary to neurological compromise. Therefore, the study of the influence of education is highly relevant for neuropsychological practice.

The absence of difference among the groups exposed to formal education on comparison of performance on the CDT according to different educational levels suggests that the CDT can be used as a cognitive screening task even in patients with low education. Further studies should investigate whether qualitative differences exist among groups exposed to formal education.

In conclusion, the results of this study showed that the CDT was especially difficult for illiterate individuals, who had lower scores.

Thus, the test appears suitable for screening visuoconstructional praxis and providing an overall impression of cognitive function among individuals, irrespective of years of education, but results should be interpreted with caution, especially among illiterate individuals.
